# The impact of the cumulative dose of cisplatin during concurrent chemoradiotherapy on the clinical outcomes of patients with advanced-stage nasopharyngeal carcinoma in an era of intensity-modulated radiotherapy

**DOI:** 10.1186/s12885-015-1964-8

**Published:** 2015-12-16

**Authors:** Shan-Shan Guo, Lin-Quan Tang, Lu Zhang, Qiu-Yan Chen, Li-Ting Liu, Ling Guo, Hao-Yuan Mo, Dong-Hua Luo, Pei-Yu Huang, Yan-Qun Xiang, Rui Sun, Ming-Yuan Chen, Lin Wang, Xing Lv, Chong Zhao, Xiang Guo, Ka-Jia Cao, Chao-Nan Qian, Mu-Sheng Zeng, Jin-Xin Bei, Ming-Huang Hong, Jian-Yong Shao, Ying Sun, Jun Ma, Hai-Qiang Mai

**Affiliations:** 1 Sun Yat-sen University Cancer Center, State Key Laboratory of Oncology in South China, Collaborative Innovation Center for Cancer Medicine, Guangzhou, China; 2 Department of Nasopharyngeal Carcinoma, Sun Yat-sen University Cancer Center, 651 Dongfeng Road East, Guangzhou, 510060 P. R. China; 3 Good Clinical Practice Center, Sun Yat-sen University Cancer Center, Guangzhou, China; 4 Department of Molecular Diagnostics, Sun Yat-sen University Cancer Center, Guangzhou, China; 5 Department of Radiation Oncology, Sun Yat-sen University Cancer Center, Guangzhou, China

**Keywords:** Nasopharyngeal carcinoma, Cumulative dose of cisplatin, IMRT, EBV DNA, Survival

## Abstract

**Background:**

The impact of cumulative dose of cisplatin on clinical outcomes of nasopharyngeal carcinoma (NPC) patients who received intensity-modulated radiotherapy (IMRT) was evaluated.

**Methods:**

This study included 491 consecutive patients with histologically confirmed NPC who were treated with concurrent chemoradiotherapy with IMRT. The patients were divided into three groups: low- (cumulative dose ≤100 mg/m^2^), medium- (cumulative dose >100 mg/m^2^ and ≤200 mg/m^2^), and high- (cumulative dose >200 mg/m^2^) dose groups. Subgroups of patients included pre-treatment levels of Epstein–Barr Virus DNA (EBV DNA) <4000 copies/ml and pre-treatment EBV DNA ≥4000 copies/ml. To test for independent significance, the Kaplan–Meier with the log–rank test and the Cox proportional hazards model were used.

**Results:**

The 5-year overall survival (OS) rates of the low-, medium-, and high-dose groups were 64.1 %, 91.1 %, and 89.4 %, respectively (*P* = 0.002). Based on multivariate analysis, patients who were in the medium- and high-dose groups had compared with the low-dose group, with an odds ratio of 0.135 (95 % CI 0.045–0.405, *P* < 0.001) and 0.225 (95 % CI 0.069–0.734, *P* = 0.013), respectively. For the low-risk patients, the cumulative dose of cisplatin significantly associated with a lower OS (*P* < 0.001). The medium-dose group had reduced odds of death compared with the low-dose group, with an odds ratio of 0.062 (95 % CI 0.001–0.347, *P* = 0.002), according to multivariate analysis.

**Conclusions:**

The cumulative dose of cisplatin is associated with OS and distant metastasis-free survival (DMFS) among NPC patients who received IMRT.

**Electronic supplementary material:**

The online version of this article (doi:10.1186/s12885-015-1964-8) contains supplementary material, which is available to authorized users.

## Background

Nasopharyngeal carcinoma (NPC) is endemic in Asia, particularly the classical nonkeratinizing type. NPC differs from other head and neck cancers by its distinctly skewed geographic and ethnic distribution, its association with Epstein–Barr virus (EBV), its aggressive natural behaviour with an especially high predilection propensity for distant metastases, and special therapeutic considerations [1]. Currently, concurrent cisplatin-based chemotherapy administered during the course of radiotherapy is considered to be the standard of care for advanced NPC. Cisplatin-based regimens delivered either once per week (30–40 mg/m^2^) or once every three weeks (100 mg/m^2^) are accepted as standard practice for concurrent chemotherapy [2–4]. Meta-analyses of randomised controlled trials and phase III studies have concluded that the addition of any type of chemotherapy to definitive RT can improve clinical outcomes [3–11]. The dose intensity of chemotherapy administered during radiotherapy has been shown to have prognostic significance in NPC treatment, but these associations were mostly based on conventional two-dimensional (2D) and three-dimensional conformal techniques [12–14]. With the development of radiation techniques, there is now little controversy that intensity-modulated radiotherapy (IMRT) is preferred for the treatment of NPC, if resources permit; dosimetric studies have shown that this procedure could improve dose conformity for complex tumour targets and improve the protection of adjacent organs. Together with chemotherapy, all IMRT series have reported excellent results, with local controls exceeding 90 % and 3-year disease-free survival rates of over 80 % [15–18]. Therefore, it is of great importance to identify the optimal cumulative dose of cisplatin for concurrent chemoradiotherapy (CCRT) in patients with NPC who receive IMRT.

In this study, we aimed to compare the long-term survival outcomes of the different cumulative doses of cisplatin that were delivered concurrently with IMRT in patients with NPC. Our findings will help guide clinical CCRT treatment strategies in NPC.

## Methods

### Ethics statement

This retrospective study was approved by the Clinical Research Ethics Committee of the Sun Yat-sen University Cancer Center, and all the participants provided written informed consent before treatment. This study is in compliance with the Helsinki Declaration. Patient records were anonymized and de-identified prior to analysis.

### Patients

This study retrospectively analysed data from 491 consecutive patients with histologically confirmed NPC who were treated with concurrent chemoradiotherapy between December 2006 and December 2010 at Sun Yat-sen University Cancer Center. Inclusion criteria for the patients consisted of (1) histologically confirmed NPC by biopsy of the nasopharynx, (2) no distant metastasis, (3) no treatment prior to admission, (4) no other tumour types or serious illnesses, (5) an Eastern Cooperative Oncology Group (ECOG) performance score ≤2, (6) received radical IMRT during the course of treatment, and (7) received concurrent chemotherapy with cisplatin. In all patients, the staging workup included an MRI of the head and neck, a chest radiograph, a bone scintigraphy, and an ultrasonography of the abdominal region. Patients who received neoadjuvant chemotherapy were ineligible. All participants were restaged according to the Seventh Edition of the American Joint Committee on Cancer (AJCC) staging system. There were 42, 328, and 121 patients with stage II, III, and IVa-b disease, respectively. Patients were divided into three groups, i.e., low-dose (cumulative dose ≤100 mg/m^2^), medium-dose (100 mg/m^2^ < cumulative dose ≤ 200 mg/m^2^), and high-dose (cumulative dose >200 mg/m^2^), according to previous studies [12, 14]. Table 1 shows the clinicopathological features in the study population of 491 patients.

**Table 1:** Baseline characteristics of 491 patients with nasopharyngeal carcinoma

	Low-dose group 14(2.9 %)	Medium-dose group 378(77.0 %)	High-dose group 99(20.2 %)	*P* value
Age(yr.),				0.081
<45	7(50.0 %)	170(45.0 %)	57(57.6 %)	
≥45	7(50.0 %)	208(55.0 %)	42(42.4 %)	
Gender				0.284
Female	6(42.9 %)	103(27.2 %)	132(26.9 %)	
Male	8(57.1 %)	275(72.8 %)	359(73.1 %)	
T stage				0.960
1	1(7.1 %)	23(6.1 %)	7(7.1 %)	
2	2(14.3 %)	68(18.0 %)	15(15.2 %)	
3	7(50.0 %)	217(57.4 %)	57(57.6 %)	
4	4(28.6 %)	70(18.5 %)	20(20.3 %)	
N stage				0.306
0	2(14.3 %)	55(14.6 %)	8(8.1 %)	
1	8(57.1 %)	140(37.0 %)	47(47.5 %)	
2	4(28.6 %)	159(42.1 %)	38(38.4 %)	
3	0(0)	24(6.3 %)	6(6.1 %)	
Clinical stage				0.492
2	3(21.4 %)	30(7.9 %)	9(9.1 %)	
3	8(57.1 %)	256(67.7 %)	64(64.6 %)	
4	3(21.4 %)	92(24.3 %)	26(26.3 %)	
WHO type				0.418
2	0(0)	15(4.5 %)	7(7.6 %)	
3	12(100.0 %)	316(95.5 %)	85(92.4 %)	
ECOG score				0.859
0	0(0)	8(2.1 %)	2(2.0 %)	
1	14(100.0 %)	370(97.9 %)	97(98.0 %)	
ACE-27				0.159
0	8(57.1 %)	296(78.1 %)	78(78.8 %)	
1	5(35.7 %)	71(18.7 %)	21(21.2 %)	
2	1(7.1 %)	12(3.2 %)	0(0)	
3	0(0)	0(0)	0(0)	
EBV DNA				0.942
≥4000	8(57.1 %)	232(61.4 %)	60(60.6 %)	
<4000	6(42.9 %)	146(38.6 %)	39(39.4 %)	
RT Dose(Gy), Median(range)	68.0(68.0-70.0)	68.0(66.0-70.0)	68.0(66.0-70.0)	0.359
VCA-IgA				0.431
Positive(≥1:80)	10(71.4 %)	314(83.1 %)	79(79.8 %)	
Negative(<1:80)	4(28.6 %)	64(16.9 %)	20(20.2 %)	
EA-IgA				0.425
Positive(≥1:10)	8(57.1 %)	276(73.0 %)	72(72.7 %)	
Negative(<1:10)	6(42.9 %)	102(27.0 %)	27(27.3 %)	
Median follow-up in months(range)	46.5(3–80)	48(1–88)	53(11–86)	0.018

*Abbreviations: yr* year, *WHO* World Health Organization, *ECOG* Eastern Cooperative Oncology Group, *EBV DNA* Epstein–Barr virus deoxyribonucleic acid, *RT* radiotherapy

*P* value < 0.05 indicates a statistically significant difference

### Treatment

The target volumes were delineated using a previously described institutional treatment protocol [19], in accordance with the International Commission on Radiation Units and Measurements reports 50 and 62. All target volumes were delineated slice-by-slice on the treatment planning computed tomography scan. The primary nasopharyngeal gross tumour volume (GTVnx) and the involved cervical lymph nodes were determined based on the imaging, clinical, and endoscopic findings. The enlarged retropharyngeal nodes were outlined, together with primary gross tumour volume (GTV), as the GTVnx on the IMRT plans. The first clinical tumour volume (CTV1) was defined as the area from 0.5 to 1.0 cm outside the GTV, a site that involves potential sites of local infiltration. Clinical target volume 2 (CTV2) was defined as the margin from 0.5 to 1.0 cm around CTV1 and the lymph node draining area (Levels II, III, and IV). For stage N1–3 patients, the lower neck area received conventional anterior cervical field radiation with a midline shield to 50 Gy in daily fractions of 2 Gy. For patients with stage N0 disease, RT was not delivered to the lower neck area. The prescribed dose was 66–70 Gy to the planning target volume (PTV) of GTVnx (PTVnx), 60 Gy to PTV1, 54 Gy to PTV2, and 60–66 Gy to PTV of the involved cervical lymph nodes in 30 to 33 fractions. In total, 30–33 fractions were administered at 1 fraction per day, 5 days/week. The IMRT plan was designed in accordance with previous studies conducted at the Sun Yat-sen University Cancer Center [20, 21].

Concurrent cisplatin chemotherapy was delivered to all of the patients. Chemotherapy was initiated on the same day as IMRT, and the cisplatin regimen included intravenous infusion (IV) of 80–100 mg/m^2^ cisplatin every 3 weeks or of 30–40 mg/m^2^ IV cisplatin weekly. Among all of the 491 patients, 14 (2.9 %) had a cumulative dose of cisplatin less than or equal to 100 mg/m^2^, 378 (77.0 %) had a cumulative dose of cisplatin >100 and ≤200 mg/m^2^, and 99 (20.2 %) had a cumulative dose of cisplatin more than 200 mg/m^2^ during treatment.

### Follow-up

The follow-up duration was calculated from the first day of treatment to either the day of death or the day of the last examination. Patients were examined at least every 3 months during the first 2 years; thereafter, follow-up examinations were performed every 6 months for 3 years or until death. The median follow-up period for the entire patient cohort was 49 months (range 1–88 months).

### Statistical analysis

The program Statistical Package for Social Sciences version 17 (SPSS Inc., Chicago, IL, USA) was used for analysis. The Kruskal–Wallis test and Fisher’s exact test were used to analyse the relationship among the low- (cumulative dose ≤100 mg/m^2^), medium- (100 mg/m^2^ < cumulative dose ≤ 200 mg/m^2^), and high- (cumulative dose > 200 mg/m^2^) dose groups among all of the NPC patients. Survival curves were estimated using the product limit method of Kaplan–Meier with the log-rank test. Univariate analysis was conducted using the log-rank test, and multivariate analyses were calculated using the Cox proportional hazards regression model. The potentially important prognostic factors considered in the modelling process included the following: patient gender (1. female, 2. male), age (1. <45, 2. ≥45), T stage (1. T1, 2. T2, 3. T3, 4. T4), N stage (1. N0, 2. N1, 3. N2, 4. N3), Epstein–Barr virus deoxyribonucleic acid (EBV DNA) (1. <4000, 2. ≥4000), and cumulative dose of cisplatin (1. low-, 2. medium-, 3. high-dose group). The entire patient cohort was divided into high- and low-risk patients by pre-treatment with EBV DNA using a cut-off value of 4000 copies/ml, according to previous studies, which led to a distinct risk stratification [22, 23]. The following end-points (time to the first defining event) were assessed: overall survival (OS), disease-free survival (DFS, distant metastasis-free survival (DMFS), and locoregional relapse-free survival (LRFS). The OS was defined as the time from diagnosis of NPC to death from any cause or until the date of the last follow-up. DFS was defined as the time from the diagnosis of NPC to events that included death or disease progression at local, regional, or distant sites or until the date of the last follow-up. LRFS was defined as the time from the diagnosis of NPC to the absence of a primary site or neck lymph node relapse or until the date of the last follow-up. DMFS was defined as the time from the date of treatment to the date of the first observation of a distant metastases or until the date of the last follow-up. The primary endpoint was OS, and secondary endpoints were DFS, DMFS, and LRFS.

Multivariate analyses using the Cox proportional hazards regression model were utilised to test for independent significance. All *P* values were two-tailed; *P* ≤ 0.05 was considered statistically significant. Our report adheres to STROBE guidelines (http://www.strobe-statement.org/) for reporting observational research (Additional file 1).

## Results

In total, 22/491 (4.5 %) patients developed locoregional failure, 53/491 (10.8 %) patients developed distant metastases, 39/491 (7.9 %) patients died, and 70/491 (14.3 %) patients developed both locoregional recurrences and distant metastases. For the entire cohort, the 5-year OS, DFS, DMFS, and LRFS rates were 90.1 %, 84.1 %, 88.2 %, and 94.8 %, respectively.

### The clinical characteristics and prognosis impact of cumulative doses of cisplatin

**Fig. 1:**
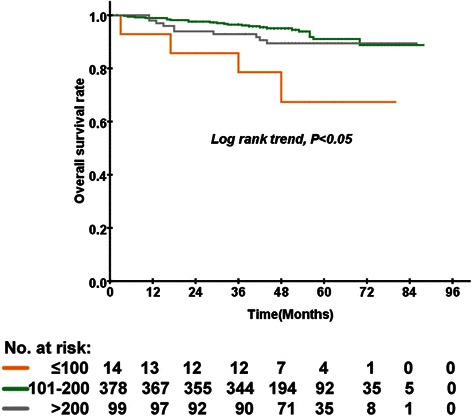
Kaplan–Meier curves of overall survival according to the cumulative dose of cisplatin in 491 patients with locally advanced nasopharyngeal carcinoma

NPC patients received low- (≤100 mg/m^2^), medium- (101–200 mg/m^2^), or high-doses (>200 mg/m^2^) of cumulative cisplatin. The clinical characteristics and treatment factors for the three groups (≤100 mg/m^2^, 101–200 mg/m^2^, >200 mg/m^2^) were well balanced. The 5-year OS rates of the low-, medium-, and high-dose groups were 64.1 %, 91.1 %, and 89.4 %, respectively (*P* = 0.002; Fig. 1). Multivariate analysis using the Cox proportional hazards regression model demonstrated that the cumulative dose of cisplatin was significantly associated with OS (Table 2), and the N stage was an independent prognostic factor for OS. Patients who were in the medium- and high-dose groups had lower odds of death than did the patients in the low-dose group, with odds ratios of 0.135 (95 % confidence intervals (CI) 0.045–0.405, *P* < 0.001) and 0.225 (95 % CI 0.069–0.734, *P* = 0.013), respectively. In addition, a significant difference in OS was observed on the N stage and EBV DNA. Patients with a N3 stage and EBV DNA ≥4000 copies/ml had an increased odd of death, with odds ratios of 7.404 (95 % CI 1.494–36.684, *P* = 0.014) and 4.953 (95 % CI 2.200–11.153, *P* < 0.001), respectively.

**Table 2:** Multivariate analysis of prognostic factors in 491 nasopharyngeal carcinoma patients receiving IMRT

Endpoint	Variable	HR	HR (95 % CI)	*P* value
OS	N Stage(0)	Ref	Ref	0.001
	N Stage(1)	1.172	0.252- 5.453	0.839
	N Stage(2)	1.767	0.393- 7.950	0.458
	N Stage(3)	7.404	1.494- 36.684	0.014
	Low-dose group	Ref	Ref	0.001
	Median-dose group	0.135	0.045-0.405	<0.001
	High-dose group	0.225	0.069-0.734	0.013
	EBV DNA	4.953	2.200-11.153	<0.001
DMFS	EBV DNA	3.669	2.058-6.540	<0.001

*HR* hazard ratio, *CI* confidence interval, *Ref* reference, *OS* overall survival, *DMFS* distant metastasis free survival, *EBV DNA* Epstein–Barr virus deoxyribonucleic acid. *P* value < 0.05 is statistically significant

**Fig. 2:**
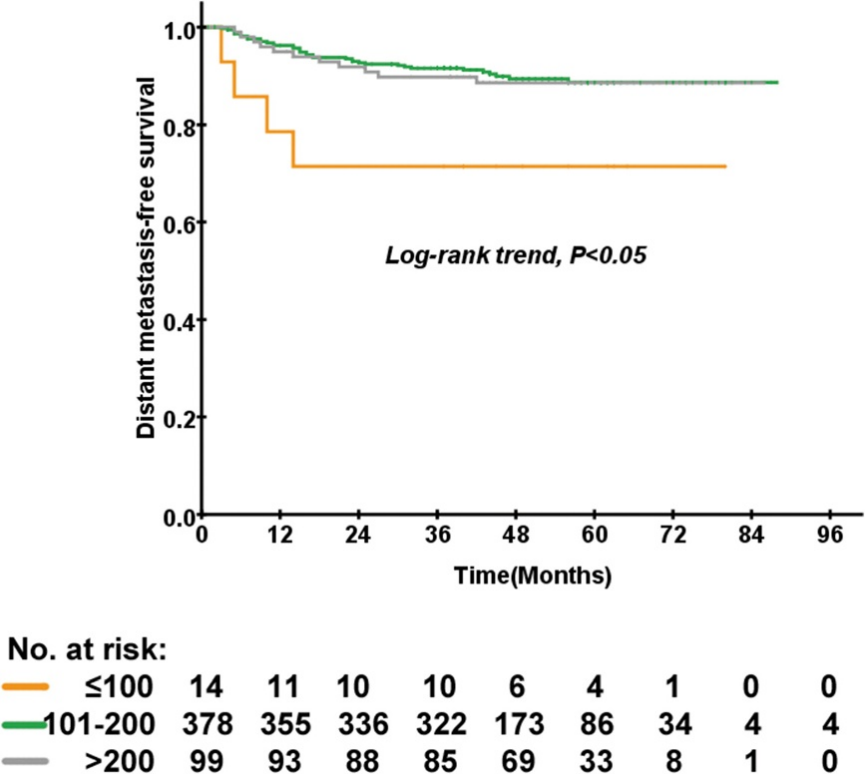
Kaplan–Meier curves of distant metastasis-free survival according to the cumulative dose of cisplatin in 491 patients with locally advanced nasopharyngeal carcinoma

The 5-year DMFS rates of the low-, medium-, and high-dose groups were 69.2 %, 88.7 %, and 88.6 %, respectively; this difference was statistically significant (*P* = 0.027; Fig. 2). Multivariate analysis using the Cox proportional hazards model demonstrated that EBV DNA was the only independent prognostic factor associated with DMFS with an OR of 3.669 (95 % CI 2.058–6.540, *P* < 0.001; Table 2). The cumulative dose of cisplatin was not significantly associated with DFS or LRFS.

### Analysis of the prognostic implications of the cumulative dose of cisplatin among all patients stratified by EBV DNA levels

**Fig. 3:**
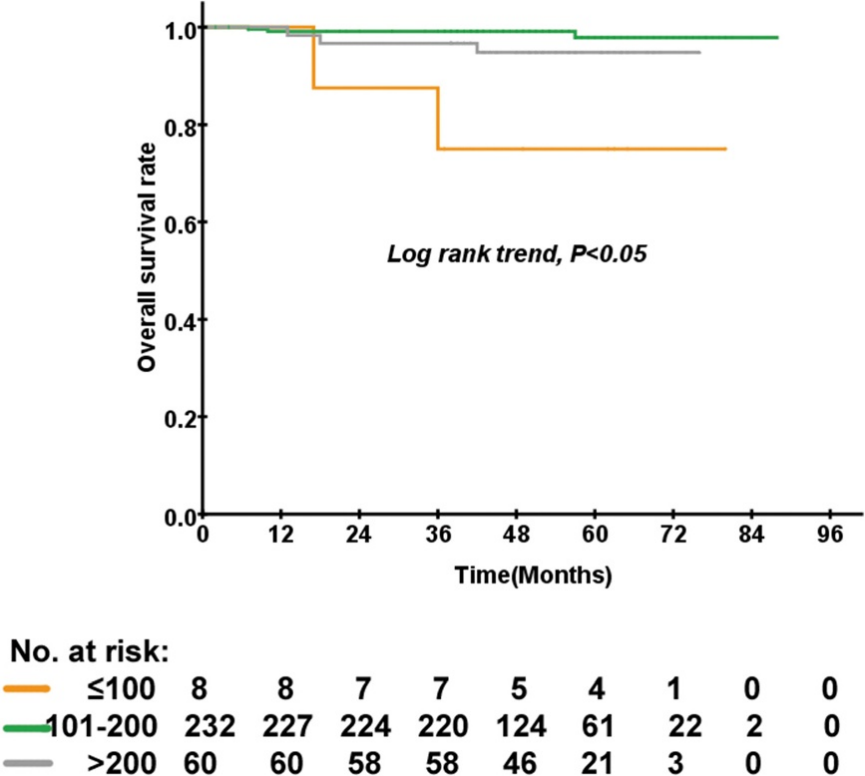
Kaplan–Meier curves of overall survival according to the cumulative dose of cisplatin in 300 low-risk patients with locally advanced nasopharyngeal carcinoma

**Fig. 4:**
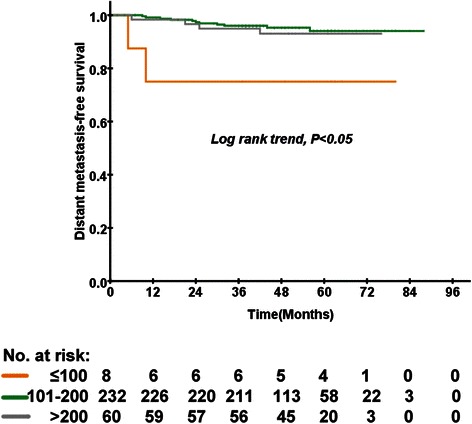
Kaplan–Meier curves of distant metastasis-free survival according to the cumulative dose of cisplatin in 300 low-risk patients with locally advanced nasopharyngeal carcinoma

There were 300 (61.1 %) and 191 (38.9 %) patients with pre-treatment EBV DNA levels less than 4000 copies/ml or EBV DNA ≥4000 copies/ml, respectively. In the low-risk group, 8 (2.7 %) patients received less than 100 mg/m^2^, 323 (77.3 %) patients received 101–200 mg/m^2^, and 60 (20.0 %) patients received more than 200 mg/m^2^. In the subgroup analysis for low-risk group patients (EBV DNA <4000 copies/ml), the cumulative dose of cisplatin was significantly associated with a lower OS based on univariate analysis (*P* < 0.001; Fig. 3). After multivariate analysis using the Cox proportional hazards regression model, the cumulative dose of cisplatin was significantly associated with OS (*P* = 0.009). The medium-dose group had reduced odds of death compared with the low-dose group, with an odds ratio of 0.062 (95 % CI 0.001–0.347, *P* = 0.002). The cumulative dose of cisplatin was significantly associated with DMFS (*P* = 0.034; Fig. 4). However, the cumulative dose of cisplatin was not significantly associated with DMFS by multivariate analysis. Moreover, the cumulative dose of cisplatin was not associated with OS or DMFS among the high-risk (EBV DNA ≥4000 copies/ml) patients by multivariate Cox regression analysis.

## Discussion

Concurrent chemotherapy combined with IMRT has been established as a standard of care for the treatment of advanced NPC because of its excellent local control and increased survival rates [24, 25]. Therefore, it is necessary to re-evaluate the impact of the cumulative dose of cisplatin on the clinical outcomes for NPC in this new era of IMRT.

Currently, to the best of our knowledge, no report has addressed the impact of the dose of cisplatin on the clinical outcomes on patients with NPC who were treated with IMRT. Several studies have analysed the impact of the dose of cisplatin on clinical outcomes of NPC using conventional 2D and 3D conformal radiotherapy technology. A previous study reported that the number of cycles of cisplatin delivered is an independent prognostic factor for OS in patients with stage II–III NPC who are undergoing concurrent chemoradiotherapy with weekly cisplatin [12]; however, this was a retrospective study that enrolled 241 patients and was mostly based on 2D or 3D conventional radiotherapy [12]. The impact of the cumulative dose of cisplatin on clinical outcomes of NPC remains unknown in this era of IMRT.

Wei et al. retrospectively compared the long-term efficacy of CCRT regimens (docetaxel vs. cisplatin), the cumulative dose intensity of cisplatin (>200 vs. ≤200 mg/m^2^), and the pre-treatment plasma levels of EBV DNA for nasopharyngeal carcinoma (NPC). This study showed that cumulative cisplatin >200 mg/m^2^ improved the 5-year PFS rates and significantly improved distant failure-free survival compared with cumulative cisplatin of ≤200 mg/m^2^ in 214 NPC patients [13]. Lee et al. reported a combined analysis of NPC-9901 and NPC-9902 Trials and found that the dose of cisplatin during the concurrent phase had a significant impact on the locoregional-failure free and OS rates; the difference between 0–1 (0–100 mg/m^2^) and 2 cycles (200 mg/m^2^) was significant [14]. The results of our study were in accordance with previous studies. Our findings suggest that the patients who received 0–100 mg/m^2^ of cisplatin had lower OS and DMFS rates than did the patients who received >100 mg/m^2^ of cisplatin concurrent chemotherapy among the 491 patients after multivariate analysis.

There was no significant difference between the patients in the medium- (101–200 mg/m^2^) and high- (>200 mg/m^2^) dose groups. Future studies are needed to evaluate whether low-risk patients who receive medium-dose cisplatin chemotherapy gain the same long-term survival benefit as those who receive high-dose cisplatin chemotherapy. In Asian populations, the rate of compliance with three cycles of cisplatin might be lower than that observed for patients in Western nations. A total of 107 (68 %) patients completed all cycles of concurrent chemotherapy in a clinical trial that compared concurrent chemoradiotherapy plus adjuvant chemotherapy with radiotherapy alone in patients with locoregionally advanced nasopharyngeal carcinoma in endemic regions of China [26].

Tao et al. retrospectively analysed 154 patients to compare the long-term survival and the toxicity of cisplatin delivered weekly versus every three weeks concurrently with IMRT in NPC. In the group receiving cisplatin every three weeks, 88.9 % of the patients completed two cycles of cisplatin, and only 6.2 % of the patients received three cycles of cisplatin. In the weekly group, 90.4 % of the patients received at least five weeks of cisplatin, and 5.5 % of the patients received seven weeks of cisplatin [2]. Further clinical studies are required to investigate the balance between clinical outcomes and the compliance with concurrent cisplatin chemotherapy and thereby identify the appropriate dose of concurrent cisplatin that is required to improve NPC patient outcomes.

We observed that the cumulative dose of cisplatin affected the overall survival and distant failure rates but did not affect local failure rates in NPC patients treated with IMRT. The main reason for this finding could be the excellent dose coverage of the locoregional site that is provided by IMRT. Indeed, the increasingly widespread use of IMRT technology in NPC patients in recent decades has improved treatment outcomes compared with conventional radiotherapy, particularly for local disease control [27–29]. The patterns of failure after IMRT predominantly result from distant metastases rather than local control. Therefore, the optimal cisplatin dose in CCRT regimes for NPC warrants further exploration. It is possible that combined use of induction chemotherapy or adjuvant chemotherapy with cisplatin-based CCRT results in reducing the DMFS rate on NPC patients treated with IMRT. Although the answer for this question is still unclear, the results of ongoing trials are expected to point out the benefits on DMFS by using induction chemotherapy or adjuvant chemotherapy combined with CCRT compared to cisplatin-based CCRT alone.

In our subgroup analysis of low-risk patients, the cumulative dose of cisplatin had an impact on OS based on the multivariate analysis. The results of the subgroup analysis of low-risk patients were in accordance with the results obtained for all patients. The low-risk group, which received a dose of cisplatin ≤ 100 mg/m^2^, was composed of only 8 patients, and therefore could be biased. In the low-risk group, only 2 patients died. Both of the patients died from distant metastases. There is an unavoidable bias because of the small sample size in the low-risk group receiving a dose of cisplatin ≤100 mg/m^2^. However, there was no difference in the clinical outcomes among the low-, medium-, and high-dose groups for the high-risk patients. We speculate that the number of high-risk patients was too small to detect any significant differences among the groups. There were 191 high-risk patients in our study, which is not a very large sample size. Thus, there is possible bias due to the small sample size in the high-risk patient group. Increasing the sample size in future studies will enable the further evaluation of the cumulative dose of cisplatin among high-risk patients with NPC with reduced bias. In addition, the high-risk patients were usually with high tumour burden, which probably progressing to tumour distant metastasis. Therefore, for these high-risk patients, concurrent chemoradiotherapy may not be very effective.

The major drawback of this study is the limitations due to the retrospective design. For example, the number of patients in the low-dose group was too small. And the study included patients who received a three-week regimen or a weekly regimen of cisplatin, which leads to possible bias. We did not provide a suggestive cisplatin delivery regimen or the optimal cumulative cisplatin dose in this study. Further studies are needed to confirm the optimal cumulative cisplatin dose and the preferred delivery cisplatin regimen. In addition, it was a single-centre study; therefore, these results need to be validated in other data sets.

## Conclusions

The cumulative dose of cisplatin is significantly associated with reduced OS and DMFS in patients who received IMRT. The findings of this study are important for further investigations into the appropriate cumulative dose of cisplatin as a concurrent chemotherapy administered in CCRT.

## Additional file

Additional file 1: **STROBE Statement—checklist of items that should be included in reports of observational studies.** (DOCX 40 kb)

## Abbreviations

NPC: Nasopharyngeal carcinoma
EBV: Epstein–Barr virus
RT: Radiotherapy
2D: Two-dimensional
IMRT: Intensity-modulated radiotherapy
CCRT: Concurrent chemoradiotherapy
ECOG: Eastern Cooperative Oncology Group
ACE-27: Adult Comorbidity Evaluation-27
AJCC: American Joint Committee on Cancer
GTVnx: Primary nasopharyngeal gross tumour volume
GTV: Primary gross tumour volume
CTV1: First clinical tumour volume
CTV2: Clinical target volume 2
PTV: Planning target volume
IV: Intravenous infusion
EBV DNA: Epstein–Barr virus deoxyribonucleic acid
OS: Overall survival
DFS: Disease-free survival
DMFS: Distant metastasis-free survival
LRFS: Locoregional relapse-free survival
3D: Three-dimensional
