# Supplementary material for: The impact of the cumulative dose of cisplatin during concurrent chemoradiotherapy on the clinical outcomes of patients with advanced-stage nasopharyngeal carcinoma in an era of intensity-modulated radiotherapy
Source: BMC Cancer. 2015 Dec 16;15:977. doi: 10.1186/s12885-015-1964-8 (PMC4682221; doi:10.1186/s12885-015-1964-8)
Supplement: Additional file 1: — STROBE Statement—checklist of items that should be included in reports of observational studies. (DOCX 40 kb) [file 12885_2015_1964_MOESM1_ESM.docx]

STROBE Statement—checklist of items that should be included in reports of observational studies

|  | Item No. | Recommendation | Page  No. | Relevant text from manuscript |
| --- | --- | --- | --- | --- |
| **Title and abstract** | 1 | (*a*) Indicate the study’s design with a commonly used term in the title or the abstract | 1-5 | The impact of the cumulative dose of cisplatin during concurrent chemoradiotherapy on the clinical outcomes of patients with advanced-stage nasopharyngeal carcinoma in an era of intensity-modulated radiotherapy |
|  |  | (*b*) Provide in the abstract an informative and balanced summary of what was done and what was found | 4-5 | Background: The impact of cumulative dose of cisplatin on clinical outcomes of nasopharyngeal carcinoma (NPC) patients who received intensity-modulated radiotherapy (IMRT) was evaluated.  Methods: This study included 491 consecutive patients with histologically confirmed NPC who were treated with concurrent chemoradiotherapy with IMRT. The patients were divided into three groups: low- (cumulative dose ≤100 mg/m^2^), medium- (cumulative dose >100 mg/m^2^ and ≤200 mg/m^2^), and high- (cumulative dose >200 mg/m2) dose groups. Subgroups of patients included pre-treatment levels of Epstein–Barr Virus DNA (EBV DNA) <4000 copies/ml and pre-treatment EBV DNA ≥4000 copies/ml. To test for independent significance, the Kaplan–Meier with the log–rank test and the Cox proportional hazards model were used.  Results: The 5-year overall survival (OS) rates of the low-, medium-, and high-dose groups were 64.1%, 91.1%, and 89.4%, respectively (P=0.002). Based on multivariate analysis, patients who were in the medium- and high-dose groups had compared with the low-dose group, with an odds ratio of 0.135 (95%CI 0.045–0.405, P<0.001) and 0.225 (95%CI 0.069–0.734, P=0.013), respectively. For the low-risk patients, the cumulative dose of cisplatin significantly associated with a lower OS (P<0.001). The medium-dose group had reduced odds of death compared with the low-dose group, with an odds ratio of 0.062 (95%CI 0.001–0.347, P=0.002), according to multivariate analysis.  Conclusions: The cumulative dose of cisplatin is associated with OS and distant metastasis-free survival (DMFS) among NPC patients who received IMRT. |
| Introduction | | | |  |
| Background/rationale | 2 | Explain the scientific background and rationale for the investigation being reported | 6-7 | Nasopharyngeal carcinoma (NPC) is endemic in Asia, particularly the classical nonkeratinizing type. NPC differs from other head and neck cancers by its distinctly skewed geographic and ethnic distribution, its association with Epstein–Barr virus (EBV), its aggressive natural behaviour with an especially high predilection propensity for distant metastases, and special therapeutic considerations. Currently, concurrent cisplatin-based chemotherapy administered during the course of radiotherapy is considered to be the standard of care for advanced NPC. Cisplatin-based regimens delivered either once per week (30–40 mg/m2) or once every three weeks (100 mg/m2) are accepted as standard practice for concurrent chemotherapy. Meta-analyses of randomised controlled trials and phase III studies have concluded that the addition of any type of chemotherapy to definitive RT can improve clinical outcomes. The dose intensity of chemotherapy administered during radiotherapy has been shown to have prognostic significance in NPC treatment, but these associations were mostly based on conventional two-dimensional (2D) and three-dimensional conformal techniques. With the development of radiation techniques, there is now little controversy that intensity-modulated radiotherapy (IMRT) is preferred for the treatment of NPC, if resources permit; dosimetric studies have shown that this procedure could improve dose conformity for complex tumour targets and improve the protection of adjacent organs. Together with chemotherapy, all IMRT series have reported excellent results, with local controls exceeding 90% and 3-year disease-free survival rates of over 80%. Therefore, it is of great importance to identify the optimal cumulative dose of cisplatin for concurrent chemoradiotherapy (CCRT) in patients with NPC who receive IMRT. |
| Objectives | 3 | State specific objectives, including any prespecified hypotheses | 7 | In this study, we aimed to compare the long-term survival outcomes of the different cumulative doses of cisplatin that were delivered concurrently with IMRT in patients with NPC. |
| Methods | | | |  |
| Study design | 4 | Present key elements of study design early in the paper | 7 | This study retrospectively analysed data from 491 consecutive patients with histologically confirmed NPC who were treated with concurrent chemoradiotherapy between December 2006 and December 2010 at Sun Yat-sen University Cancer Center. |
| Setting | 5 | Describe the setting, locations, and relevant dates, including periods of recruitment, exposure, follow-up, and data collection | 7,10 | This study retrospectively analysed data from 491 consecutive patients with histologically confirmed NPC who were treated with concurrent chemoradiotherapy between December 2006 and December 2010 at Sun Yat-sen University Cancer Center.  Patients were examined at least every 3 months during the first 2 years; thereafter, follow-up examinations were performed every 6 months for 3 years or until death. |
| Participants | 6 | (*a*) *Cohort study*—Give the eligibility criteria, and the sources and methods of selection of participants. Describe methods of follow-up  *Case-control study*—Give the eligibility criteria, and the sources and methods of case ascertainment and control selection. Give the rationale for the choice of cases and controls  *Cross-sectional study*—Give the eligibility criteria, and the sources and methods of selection of participants | 8,10 | Inclusion criteria for the patients consisted of (1) histologically confirmed NPC by biopsy of the nasopharynx, (2) no distant metastasis, (3) no treatment prior to admission, (4) no other tumour types or serious illnesses, (5) an Eastern Cooperative Oncology Group (ECOG) performance score ≤2, (6) received radical IMRT during the course of treatment, and (7) received concurrent chemotherapy with cisplatin. In all patients, the staging workup included an MRI of the head and neck, a chest radiograph, a bone scintigraphy, and an ultrasonography of the abdominal region. Patients who received neoadjuvant chemotherapy were ineligible. All participants were restaged according to the Seventh Edition of the American Joint Committee on Cancer (AJCC) staging system.  The follow-up duration was calculated from the first day of treatment to either the day of death or the day of the last examination. Patients were examined at least every 3 months during the first 2 years; thereafter, follow-up examinations were performed every 6 months for 3 years or until death. The median follow-up period for the entire patient cohort was 49 months (range 1–88 months). |
|  |  | (*b*) *Cohort study*—For matched studies, give matching criteria and number of exposed and unexposed  *Case-control study*—For matched studies, give matching criteria and the number of controls per case |  |  |
| Variables | 7 | Clearly define all outcomes, exposures, predictors, potential confounders, and effect modifiers. Give diagnostic criteria, if applicable | 8,11 | In all patients, the staging workup included an MRI of the head and neck, a chest radiograph, a bone scintigraphy, and an ultrasonography of the abdominal region. Patients who received neoadjuvant chemotherapy were ineligible.  The following end-points (time to the first defining event) were assessed: overall survival (OS), disease-free survival (DFS, distant metastasis-free survival (DMFS), and locoregional relapse-free survival (LRFS). The OS was defined as the time from diagnosis of NPC to death from any cause or until the date of the last follow-up. DFS was defined as the time from the diagnosis of NPC to events that included death or disease progression at local, regional, or distant sites or until the date of the last follow-up. LRFS was defined as the time from the diagnosis of NPC to the absence of a primary site or neck lymph node relapse or until the date of the last follow-up. DMFS was defined as the time from the date of treatment to the date of the first observation of a distant metastases or until the date of the last follow-up. The primary endpoint was OS, and secondary endpoints were DFS, DMFS, and LRFS.  The potentially important prognostic factors considered in the modelling process included the following: patient gender (1. female, 2. male), age (1. <45, 2. ≥45), T stage (1. T1, 2. T2, 3. T3, 4. T4), N stage (1. N0, 2. N1, 3. N2, 4. N3), Epstein–Barr virus deoxyribonucleic acid (EBV DNA) (1. <4000, 2. ≥4000), and cumulative dose of cisplatin (1. low-, 2. medium-, 3. high-dose group). |
| Data sources/ measurement | 8* | For each variable of interest, give sources of data and details of methods of assessment (measurement). Describe comparability of assessment methods if there is more than one group | 9-10 | Concurrent cisplatin chemotherapy was delivered to all of the patients. Chemotherapy was initiated on the same day as IMRT, and the cisplatin regimen included intravenous infusion (IV) of 80–100 mg/m^2^ cisplatin every 3 weeks or of 30–40 mg/m^2^ IV cisplatin weekly. Among all of the 491 patients, 14 (2.9%) had a cumulative dose of cisplatin less than or equal to 100 mg/m^2^, 378 (77.0%) had a cumulative dose of cisplatin >100 and ≤200 mg/m^2^, and 99 (20.2%) had a cumulative dose of cisplatin more than 200 mg/m^2^ during treatment. |
| Bias | 9 | Describe any efforts to address potential sources of bias | 17-18 | There were 191 high-risk patients in our study, which is not a very large sample size. Thus, there is possible bias due to the small sample size in the high-risk patient group. Increasing the sample size in future studies will enable the further evaluation of the cumulative dose of cisplatin among high-risk patients with NPC with reduced bias. I |
| Study size | 10 | Explain how the study size was arrived at | NA |  |

Continued on next page

| Quantitative variables | 11 | Explain how quantitative variables were handled in the analyses. If applicable, describe which groupings were chosen and why | 8 | Patients were divided into three groups, i.e., low-dose (cumulative dose ≤100 mg/m^2^), medium-dose (100 mg/m^2^ <cumulative dose≤200 mg/m^2^), and high-dose (cumulative dose >200 mg/m^2^), according to previous studies. |
| --- | --- | --- | --- | --- |
| Statistical methods | 12 | (*a*) Describe all statistical methods, including those used to control for confounding | 10 | The Kruskal–Wallis test and Fisher’s exact test were used to analyse the relationship among the low- (cumulative dose ≤100 mg/m^2^), medium- (100 mg/m^2^ <cumulative dose≤200 mg/m^2^), and high- (cumulative dose>200 mg/m^2^) dose groups among all of the NPC patients. Survival curves were estimated using the product limit method of Kaplan–Meier with the log-rank test. Univariate analysis was conducted using the log-rank test, and multivariate analyses were calculated using the Cox proportional hazards regression model. |
|  |  | (*b*) Describe any methods used to examine subgroups and interactions | 13-14 | In the subgroup analysis for low-risk group patients (EBV DNA <4000 copies/ml), the cumulative dose of cisplatin was significantly associated with a lower OS based on univariate analysis  However, the cumulative dose of cisplatin was not significantly associated with DMFS by multivariate analysis. Moreover, the cumulative dose of cisplatin was not associated with OS or DMFS among the high-risk (EBV DNA ≥4000 copies/ml) patients by multivariate Cox regression analysis. |
|  |  | (*c*) Explain how missing data were addressed | NA |  |
|  |  | (*d*) *Cohort study*—If applicable, explain how loss to follow-up was addressed  *Case-control study*—If applicable, explain how matching of cases and controls was addressed  *Cross-sectional study*—If applicable, describe analytical methods taking account of sampling strategy | NA |  |
|  |  | (*e*) Describe any sensitivity analyses | NA |  |
| Results | | | | |
| Participants | 13* | (a) Report numbers of individuals at each stage of study—eg numbers potentially eligible, examined for eligibility, confirmed eligible, included in the study, completing follow-up, and analysed | 7-10 | This study retrospectively analysed data from 491 consecutive patients with histologically confirmed NPC who were treated with concurrent chemoradiotherapy between December 2006 and December 2010 at Sun Yat-sen University Cancer Center.  There were 42, 328, and 121 patients with stage II, III, and IVa-b disease, respectively.  Among all of the 491 patients, 14 (2.9%) had a cumulative dose of cisplatin less than or equal to 100 mg/m^2^, 378 (77.0%) had a cumulative dose of cisplatin >100 and ≤200 mg/m^2^, and 99 (20.2%) had a cumulative dose of cisplatin more than 200 mg/m^2^ during treatment. |
|  |  | (b) Give reasons for non-participation at each stage |  |  |
|  |  | (c) Consider use of a flow diagram | NA |  |
| Descriptive data | 14* | (a) Give characteristics of study participants (eg demographic, clinical, social) and information on exposures and potential confounders | 10 | Among all of the 491 patients, 14 (2.9%) had a cumulative dose of cisplatin less than or equal to 100 mg/m^2^, 378 (77.0%) had a cumulative dose of cisplatin >100 and ≤200 mg/m^2^, and 99 (20.2%) had a cumulative dose of cisplatin more than 200 mg/m^2^ during treatment. |
|  |  | (b) Indicate number of participants with missing data for each variable of interest | NA |  |
|  |  | (c) *Cohort study*—Summarise follow-up time (eg, average and total amount) | 10 | The median follow-up period for the entire patient cohort was 49 months (range 1–88 months). |
| Outcome data | 15* | *Cohort study*—Report numbers of outcome events or summary measures over time | 12 | In total, 22/491 (4.5%) patients developed locoregional failure, 53/491 (10.8%) patients developed distant metastases, 39/491 (7.9%) patients died, and 70/491 (14.3%) patients developed both locoregional recurrences and distant metastases. |
|  |  | *Case-control study—*Report numbers in each exposure category, or summary measures of exposure |  |  |
|  |  | *Cross-sectional study—*Report numbers of outcome events or summary measures |  |  |
| Main results | 16 | (*a*) Give unadjusted estimates and, if applicable, confounder-adjusted estimates and their precision (eg, 95% confidence interval). Make clear which confounders were adjusted for and why they were included | 10-12 | Univariate analysis was conducted using the log-rank test, and multivariate analyses were calculated using the Cox proportional hazards regression model. The potentially important prognostic factors considered in the modelling process included the following: patient gender (1. female, 2. male), age (1. <45, 2. ≥45), T stage (1. T1, 2. T2, 3. T3, 4. T4), N stage (1. N0, 2. N1, 3. N2, 4. N3), Epstein–Barr virus deoxyribonucleic acid (EBV DNA) (1. <4000, 2. ≥4000), and cumulative dose of cisplatin (1. low-, 2. medium-, 3. high-dose group). The 5-year OS rates of the low-, medium-, and high-dose groups were 64.1%, 91.1%, and 89.4%, respectively (P=0.002; Figure 1). Multivariate analysis using the Cox proportional hazards regression model demonstrated that the cumulative dose of cisplatin was significantly associated with OS (Table 2), and the N stage was an independent prognostic factor for OS. Patients who were in the medium- and high-dose groups had lower odds of death than did the patients in the low-dose group, with odds ratios of 0.135 (95% confidence intervals (CI) 0.045–0.405, P<0.001) and 0.225 (95%CI 0.069–0.734, P=0.013), respectively. In addition, a significant difference in OS was observed on the N stage and EBV DNA. Patients with a N3 stage and EBV DNA ≥4000 copies/ml had an increased odd of death, with odds ratios of 7.404 (95%CI 1.494–36.684, P=0.014) and 4.953 (95%CI 2.200–11.153, P<0.001), respectively. |
|  |  | (*b*) Report category boundaries when continuous variables were categorized | 8,11 | Patients were divided into three groups, i.e., low-dose (cumulative dose ≤100 mg/m^2^), medium-dose (100 mg/m^2^ <cumulative dose≤200 mg/m^2^), and high-dose (cumulative dose >200 mg/m^2^), according to previous studies  The entire patient cohort was divided into high- and low-risk patients by pre-treatment with EBV DNA using a cut-off value of 4000 copies/ml, according to previous studies, which led to a distinct risk stratification |
|  |  | (*c*) If relevant, consider translating estimates of relative risk into absolute risk for a meaningful time period |  |  |

Continued on next page

| Other analyses | 17 | Report other analyses done—eg analyses of subgroups and interactions, and sensitivity analyses | 13-14 | There were 300 (61.1%) and 191 (38.9%) patients with pre-treatment EBV DNA levels less than 4000 copies/ml or EBV DNA ≥4000 copies/ml, respectively. In the low-risk group, 8 (2.7%) patients received less than 100 mg/m2, 323 (77.3%) patients received 101–200 mg/m^2^, and 60 (20.0%) patients received more than 200 mg/m^2^. In the subgroup analysis for low-risk group patients (EBV DNA <4000 copies/ml), the cumulative dose of cisplatin was significantly associated with a lower OS based on univariate analysis (P<0.001; Figure 3). After multivariate analysis using the Cox proportional hazards regression model, the cumulative dose of cisplatin was significantly associated with OS (P=0.009).The medium-dose group had reduced odds of death compared with the low-dose group, with an odds ratio of 0.062 (95%CI 0.001–0.347, P=0.002). The cumulative dose of cisplatin was significantly associated with DMFS (P=0.034; Figure 4). However, the cumulative dose of cisplatin was not significantly associated with DMFS by multivariate analysis. Moreover, the cumulative dose of cisplatin was not associated with OS or DMFS among the high-risk (EBV DNA ≥4000 copies/ml) patients by multivariate Cox regression analysis. |
| --- | --- | --- | --- | --- |
| Discussion | | | | |
| Key results | 18 | Summarise key results with reference to study objectives | 15 | Our findings suggest that the patients who received 0–100 mg/m^2^ of cisplatin had lower OS and DMFS rates than did the patients who received >100 mg/m^2^ of cisplatin concurrent chemotherapy among the 491 patients after multivariate analysis.  There was no significant difference between the patients in the medium- (101–200 mg/m^2^) and high- (>200 mg/m^2^) dose groups. |
| Limitations | 19 | Discuss limitations of the study, taking into account sources of potential bias or imprecision. Discuss both direction and magnitude of any potential bias | 18 | The major drawback of this study is the limitations due to the retrospective design. For example, the number of patients in the low-dose group was too small. And the study included patients who received a three-week regimen or a weekly regimen of cisplatin, which leads to possible bias. We did not provide a suggestive cisplatin delivery regimen or the optimal cumulative cisplatin dose in this study. Further studies are needed to confirm the optimal cumulative cisplatin dose and the preferred delivery cisplatin regimen. In addition, it was a single-centre study; therefore, these results need to be validated in other data sets. |
| Interpretation | 20 | Give a cautious overall interpretation of results considering objectives, limitations, multiplicity of analyses, results from similar studies, and other relevant evidence | 14-18 | Wei et al. retrospectively compared the long-term efficacy of CCRT regimens (docetaxel vs. cisplatin), the cumulative dose intensity of cisplatin (>200 vs. ≤200 mg/m^2^), and the pre-treatment plasma levels of EBV DNA for nasopharyngeal carcinoma (NPC). This study showed that cumulative cisplatin >200 mg/m^2^ improved the 5-year PFS rates and significantly improved distant failure-free survival compared with cumulative cisplatin of ≤200 mg/m^2^ in 214 NPC patients. Lee et al. reported a combined analysis of NPC-9901 and NPC-9902 Trials and found that the dose of cisplatin during the concurrent phase had a significant impact on the locoregional-failure free and OS rates; the difference between 0–1 (0-100 mg/m^2^) and 2 cycles (200 mg/m^2^) was significant. The results of our study were in accordance with previous studies.  We observed that the cumulative dose of cisplatin affected the overall survival and distant failure rates but did not affect local failure rates in NPC patients treated with IMRT. The main reason for this finding could be the excellent dose coverage of the locoregional site that is provided by IMRT. Indeed, the increasingly widespread use of IMRT technology in NPC patients in recent decades has improved treatment outcomes compared with conventional radiotherapy, particularly for local disease control. The patterns of failure after IMRT predominantly result from distant metastases rather than local control. Therefore, the optimal cisplatin dose in CCRT regimes for NPC warrants further exploration. It is possible that combined use of induction chemotherapy or adjuvant chemotherapy with cisplatin-based CCRT results in reducing the DMFS rate on NPC patients treated with IMRT. Although the answer for this question is still unclear, the results of ongoing trials are expected to point out the benefits on DMFS by using induction chemotherapy or adjuvant chemotherapy combined with CCRT compared to cisplatin-based CCRT alone. |
| Generalisability | 21 | Discuss the generalisability (external validity) of the study results | 17 | Therefore, the optimal cisplatin dose in CCRT regimes for NPC warrants further exploration. It is possible that combined use of induction chemotherapy or adjuvant chemotherapy with cisplatin-based CCRT results in reducing the DMFS rate on NPC patients treated with IMRT. Although the answer for this question is still unclear, the results of ongoing trials are expected to point out the benefits on DMFS by using induction chemotherapy or adjuvant chemotherapy combined with CCRT compared to cisplatin-based CCRT alone. |
| Other information | |  | | |
| Funding | 22 | Give the source of funding and the role of the funders for the present study and, if applicable, for the original study on which the present article is based | 20 | This study was supported by grants from the National Science Foundation for Distinguished Young Scholars of China (Grant No. 81425018), National Natural Science Foundation of China (No. 81072226), the 863 Project (No. 2012AA02A501), the National Key Basic Research Program of China (No.2013CB910304), the Sci-Tech Project Foundation of Guangdong Province (No.2011B080701034), the Sci-Tech Project Foundation of Guangzhou City (No.2011J4300100), the Sun Yat-sen University Clinical Research 5010 Program, and the Fundamental Research Funds for the Central Universities. |

*Give information separately for cases and controls in case-control studies and, if applicable, for exposed and unexposed groups in cohort and cross-sectional studies.

**Note:** An Explanation and Elaboration article discusses each checklist item and gives methodological background and published examples of transparent reporting. The STROBE checklist is best used in conjunction with this article (freely available on the Web sites of PLoS Medicine at http://www.plosmedicine.org/, Annals of Internal Medicine at http://www.annals.org/, and Epidemiology at http://www.epidem.com/). Information on the STROBE Initiative is available at www.strobe-statement.org.
